# Permeation Properties and Pore Structure of Surface Layer of Fly Ash Concrete

**DOI:** 10.3390/ma7064282

**Published:** 2014-05-30

**Authors:** Jun Liu, Qiwen Qiu, Feng Xing, Dong Pan

**Affiliations:** Guangdong Provincial Key Laboratory of Durability for Marine Civil Engineering, Shenzhen University, Shenzhen 518060, Guangdong, China; E-Mails: liujun@szu.edu.cn (J.L.); 2011090158@email.szu.edu.cn (D.P.)

**Keywords:** permeation properties, surface layer, fly ash, concrete, chloride immersion, water permeability, pore structure

## Abstract

This paper presents an experimental study on the nature of permeation properties and pore structure of concrete surface layers containing fly ash. Concretes containing different dosages of fly ash as a replacement for cement (15% and 30% by weight of total cement materials, respectively) were investigated. Concrete without any fly ash added was also employed as the reference specimen. Laboratory tests were conducted to determine the surface layer properties of concrete including chloride transport, apparent water permeability and pore structure. The results demonstrate that incorporation of fly ash, for the early test period, promotes the chloride ingress at the surface layer of concrete but substituting proportions of fly ash may have little impact on it. With the process of chloride immersion, the chloride concentration at the surface layer of concrete with or without fly ash was found to be nearly the same. In addition, it is suggested that the water permeability at the concrete surface area is closely related to the fly ash contents as well as the chloride exposure time. Pore structure was characterized by means of mercury intrusion porosimetry (MIP) test and the scanning electron microscopy (SEM) images. The modification of pore structure of concrete submersed in distilled water is determined by the pozzolanic reaction of fly ash and the calcium leaching effect. The pozzolanic reaction was more dominant at the immersion time of 180 days while the calcium leaching effect became more evident after 270 days.

## 1. Introduction

When concrete is in long-term contact with the potentially aggressive aqueous environments, chemical changes (often starts at surface area of the mixture) may take place [1,2]. It is generally recognized that the concrete surroundings can be classified into the air environment and the marine environment. When the concrete structure is exposed to marine environment, fluid transport takes place from the surface area of concrete to the interior matrix due to a hydrostatic pressure gradient. If that, chloride ion penetration and subsequent corrosion of embedded steel bars through a depassivation process may occur [3]. In view of the fact that aggressive ions tend to initially accumulate at the surface of concrete before penetrating into the matrix, the surface layer of concrete plays a vitally protective role in the structure. In the studies of researchers [4,5], the surface layer is greatly linked to transport properties of concrete. In this regard, understanding of the properties of surface layer in concrete is a crucial step for long term durability design of concrete structure.

It is generally acknowledged that cement is an important and integral segment of concrete materials. However, the overproduction of cement introduces high hydration heat, high autogenous shrinkage and high cost [6]. Fly ash is a by-product of coal combustion in power plants that can be used in concrete as a cement replacement [7,8,9,10]. Fly ash has demonstrated to be an attractive alternative for partial substitution of cement as it can overcome the shortcomings in manufacturing of cement with the following aspects: (1) In mass concrete applications, it can reduce the heat from hydration which results in the micro-cracks [11,12]; (2) Reduction of greenhouse gas emission during production and application [13,14,15]; (3) The fine fly ash with spherical particles and smooth surface cuts down the water requirement of concrete [16], which improves the long-term workability; (4) Fly ash could refine the pore structure of the paste by the pozzolanic reaction [17,18], which benefits the transport properties and durability of the cement paste or concrete [19,20]. Fly ash has been considered as one of the integral components of the worldwide concrete industry.

Numerous investigations regarding the pore structure or permeability of fly ash concrete have been carried out by many researchers in the past. In a study undertaken by Zhuqing Yu [21], it was shown that the total porosity of cement paste blended with fly ash is higher than that of Portland cement paste regardless of the w/b ratio and the dosage of fly ash, under long-term curing ages up to 2 years. Jiang and Guan [22] has tested the pastes with different fly ash contents using the method of mercury intrusion. He found that the total porosity of the paste increased with increasing fly ash content while the pore size distribution of high-volume fly ash paste is different from that of neat cement paste. An investigation done by Boğa and Topçu [23] on concrete and reinforced concrete with various fly ash contents indicated that samples without fly ash additive had high chloride permeability. When fly ash was replaced by cement at a 15% ratio, it was found that all of the concretes had low chloride permeability. Thomas and Bamforth [24] developed a chloride transport model to study the impact of incorporation of fly ash on the transport properties of concrete. The study shows that the rate of reduction of diffusivity is far greater for concrete containing fly ash or slag than for plain Portland cement concrete. Besides, mechanical strength, and water and chloride permeability of alkali activated slag mortar and concretes with partial fly ash substitution was investigated by Ismail *et al.* [25]. However, efforts in the direction of the surface layer properties of fly ash concrete are still limited.

Therefore, the main objective of the present study is to explore the properties of the surface layer in concrete with certain proportions of fly ash replacement. Experimental results on chloride ion precipitation in the surface layer, water permeability coefficient and pore size distribution will be presented and discussed in subsequent sections.

## 2. Results and Discussion

### 2.1. Effects of Fly Ash on Chloride Ion Ingress in the Surface Layer of Concrete

Contents of chloride ion in the surface layer of all concrete under saturated conditions for 30, 60, 90, 120 and 150 days are shown in Table 1.

**Table 1:** Contents of chloride ion in the surface layer of concrete at different immersion time (%).

**Mix ID**	**Immersion Time (day)**				
	30	60	90	120	150
CF0	0.295	0.408	0.450	0.679	0.684
CF15	0.397	0.505	0.630	0.752	0.790
CF30	0.397	0.576	0.625	0.700	0.777

**Figure 1:**
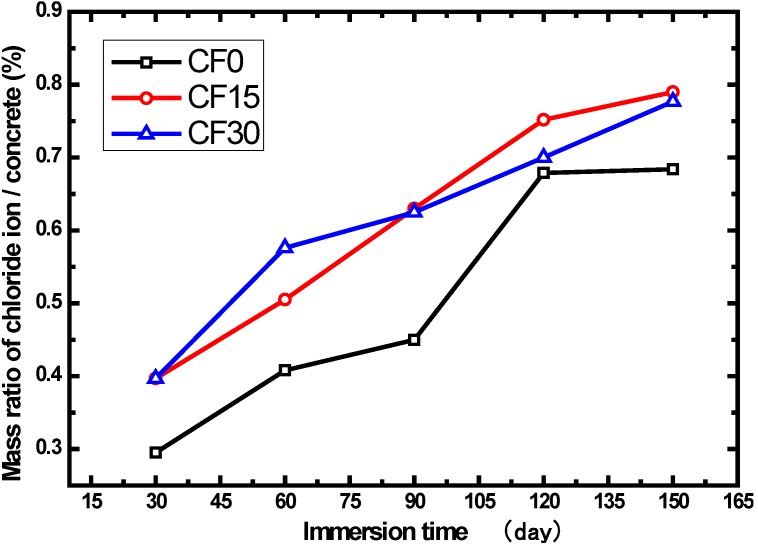
Correlation between the fly ash and chloride ion content in the surface layer of concrete.

Based on the previous study [26], the most important parameter that influences the chloride ion transport is the materials’ pore structure. The use of supplementary cementing materials (SCM) such as fly ash, silica fume, *etc.* have been found to be beneficial in resisting the ingress of chloride ions into concrete because of the microstructure densification caused by the pozzolanic reaction or secondary hydration of these materials [27]. From the results observed in Table 1 and Figure 1, it can be observed that the chloride concentration in the surface layer of fly ash concrete increases throughout the entire test period. The mixtures, respectively containing 15% and 30% fly ash replacement, show higher chloride concentrations than the reference specimen (CF0). It is mainly attributed to the lack of activated materials caused by fly ash replacement in early cement hydration, which postpones the cement hydration process. It should be noted that the ongoing and sufficient hydration reactions in Portland cement are essential to the durability and quality of concrete [28,29,30,31]. Thereby, the chloride ingress of the concrete with fly ash is far more remarkable than the concrete without any fly ash. In Figure 1, it can also be found that there is no evident difference in the chloride concentrations between CF15 and CF30, which indicates that the fly ash dosage has little impact on the chloride accumulation in the surface layer of concrete. Along with the immersion time, the chloride contents of all the samples tend to be closed. This can be explained by the fact that the pozzolanic reaction becomes more and more pronounced with increases in time, resulting in microstructure densification. Accumulation of chloride ion in the surface layer is therefore hindered. The chloride concentration in the surface layer of fly ash concrete and the ordinary Portland cement concrete (OPC) are at the same level.

### 2.2. Influence of Fly Ash on the Water Permeability in the Surface Layer

**Figure 2:**
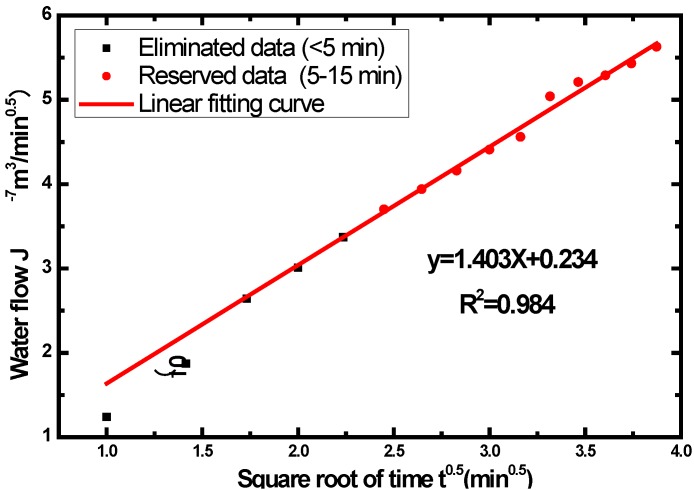
Water permeability results of concrete CF15 and their trend line.

Table 2 depicts the seepage volume of concrete CF15 sample under distilled water-eroding procedure within 15 min, by the method of Autoclam tester stated in Section 3.4. Due to the instability of the seepage in the early test period, the results data before 5 min are eliminated. Based on the results from 5 to 15 min, the seepage volume is found to be the linear function of square root time. The linear fitting curve of results of CF15 is given in Figure 2. The Table 3 summarizes the relations between each of these permeability results of CF15 at 28, 30, 60, 90, 120 and 150 days, respectively.

Surface permeability coefficient of CF0 and CF30 under ultrapure water is also provided in the following Table 4.

**Figure 3:**
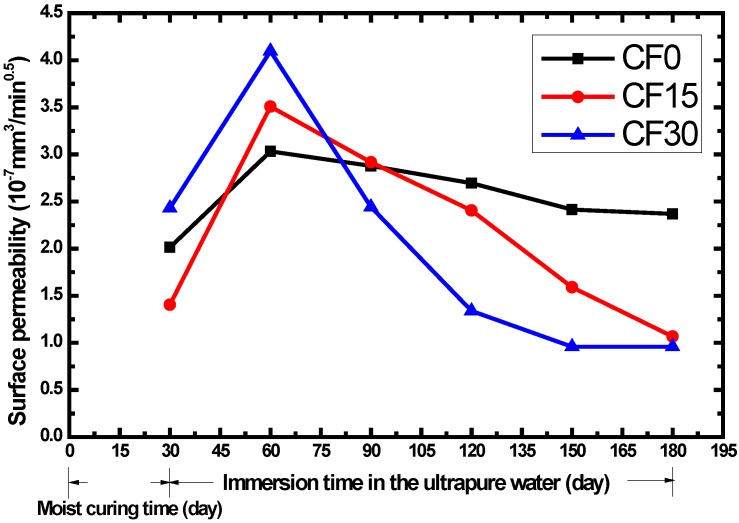
The effect of fly ash on surface permeability coefficient of concrete.

Based on the experimental data provided in Table 4, the change of surface permeability according to immersion time in distilled water is shown in Figure 3. It can be seen that fly ash has a significant effect on the water ingress in the surface layer after the curing period. At the time of 60 days, the mixture containing 30% FA replacement showed the highest surface permeability, followed by the concrete with 15% FA addition and the reference specimen, respectively. With the progress of immersion, after the age of 90 days, the case is quite different. The CF30 claims the lowest surface permeability while the reference specimen has the highest.

**Table 2:** Seepage volume of concrete CF15 sample under ultrapure water-eroding procedure within 15 min.

**Immersion time (day)**	**Water permeability test time (min)**														
	1	2	3	4	5	6	7	8	9	10	11	12	13	14	15
0	124	187	264	301	337	370	394	416	441	456	1598	521	529	543	563
30	539	805	997	1125	1246	1330	1423	1488	1547	1598	1666	1713	1756	1795	1836
60	204	353	426	550	602	654	697	799	835	870	900	930	1017	1041	1069
90	338	571	725	807	876	930	1002	1047	1086	1121	1153	1203	1229	1257	1278
120	104	154	197	253	288	318	344	370	396	422	442	463	502	520	543
150	55	89	113	139	161	182	200	221	249	262	271	295	305	319	333

**Table 3:** The fitting of the permeability coefficient of CF15.

Immersion time (day)	Fitting Curve	R^2^
0	*y* = 1.403*x* + 0.234	0.984
30	*y* = 3.509*x* + 4.893	0.996
60	*y* = 2.916*x* + 3.588	0.992
90	*y* = 2.405*x* − 0.546	0.982
120	*y* = 1.590*x* − 0.784	0.994
150	*y* = 1.067*x* − 0.786	0.994

**Table 4:** Surface permeability coefficient of concrete under distilled water-eroding at different ages.

**Sample**	**Immersion Period (day)**					
	0	30	60	90	120	150
CF0	2.014	3.033	2.879	2.694	2.414	2.369
CF15	1.403	3.509	2.916	2.405	1.590	1.067
CF30	2.429	4.098	2.442	1.339	0.958	0.958

**Figure 4:**
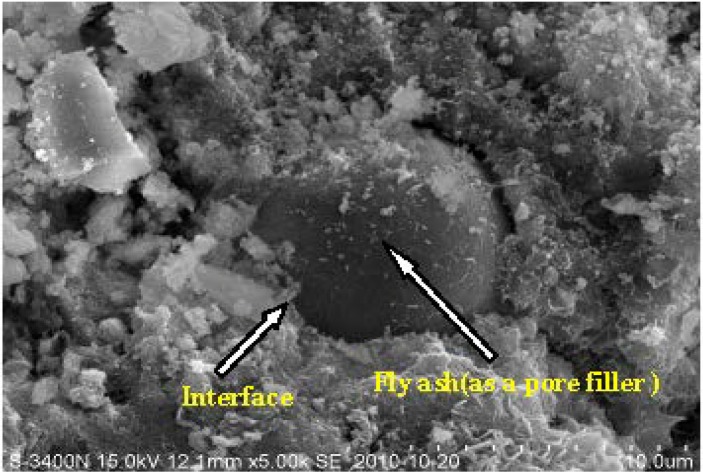
Scanning electron image of specimen C15 hydrated for 28 days showing morphology of fly ash particle.

Generally speaking, inclusion of fly ash has the following main effects: micro-filler effect and pozzolanic reaction. Pozzolanic reaction occurs from the reaction of Ca(OH)_2_ with SiO_2_ and Al_2_O_3_ from fly ash [17]. However, pozzolanic reaction of fly ash is not evident in the early hydration ages. Fly ash particles only act as the pore filler (see Figure 4). Worse still, the cement hydration is postponed by incorporation of fly ash [32]. An explanation for this is that the Ca^2+^ ions in the cement hydration system are attached and fixed to fly ash particles [33]. The reduced availability of Ca^2+^ ions prolongs the nucleation of CH or CSH. Therefore, in the early ages, due to the low levels of the Ca^2+^ ions caused by fly ash replacement, the progress of cement hydration in this period is slow and incomplete. Moreover, the delay of hydration is founded to be even more remarkable with an increase in fly ash, which hinders the pozzolanic reaction due to the lack of hydrate product Ca(OH)_2_ [34].

With the immersion process, secondary hydration of fly ash produces a large amount of CSH, which reduces the large capillary pores sizes and thus enhances the high resistance to water ingress. A series of papers have pointed out that the permeable nature of concrete goes hand in hand with the size of capillary pores [35]. Incorporation of pozzolanic materials leads to the pore structure densification of the concrete and the CSH gels/aggregates’ interface, leading to a reduction in the porosity of capillary pores, to refine the size of pores and to increase their sinuosity.

### 2.3. Influence of Fly Ash on the Pore Structure for Surface Layer of Concrete

**Figure 5:**
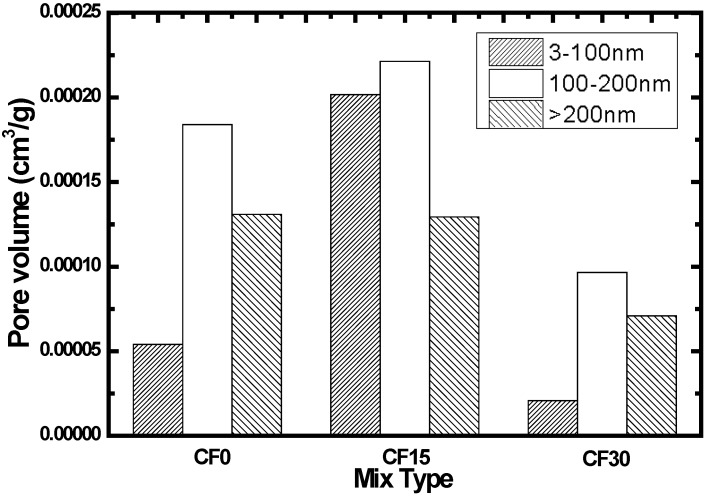
Pore size distribution of concrete sample immersed in ultrapure water at 90 days.

Results of the pore size distribution for fly ash concrete that was immersed in the distilled water at 90 days is given in Figure 5. Based on the results observed from Figure 5, the pore volume (<100 nm) of CF15 is found to increase as compared to the reference specimen (CF0), while the pore volume (>200 nm) does not show any variations. It can be explained by the presence of fly ash which leads to filling of the pores inside the concrete. As regards the specimen CF30, the pore volume (>200 nm) declined significantly compared to the CF0 and CF 15, which can be attributed to the strong activation of pozzolanic material when concrete has been immersed for 90 days. Secondary hydration must therefore occur in order to form calcium silicate hydrate. In this regard, the concrete samples become denser and in turn the impermeability is improved.

**Figure 6:**
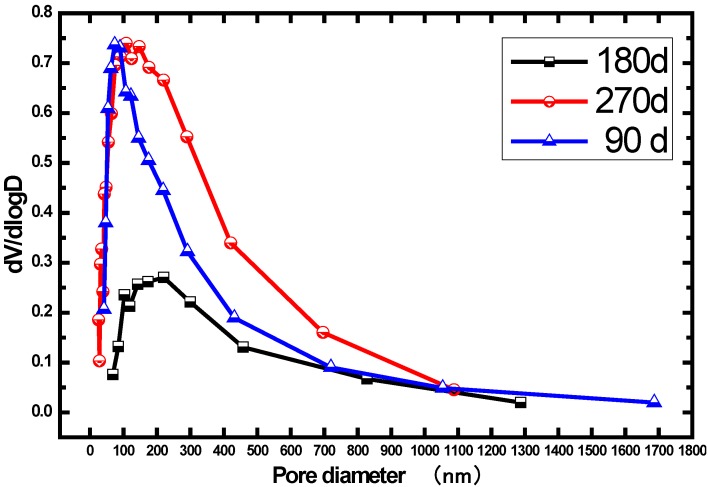
Pore size distribution of concrete CF15 sample immersed in distilled water.

Figure 6 provides us the information of pore size distribution and differential curves for CF15 at different ages. According to the studies outlined in [9,36], pores whose diameter are 100 nm are defined as threshold, which significantly determines the permeability of concrete. When the pore size exceeds the threshold, the concrete matrix tends to be more permeable [9]. Hence, the pores (> or <100 nm) are discussed in this research. At the age of 180 days, the amount of pores with all diameters (> and <100 nm) is least, in comparison to the 90 days. An explanation for this lies in the denser pore structure from the pozzolanic reaction or secondary hydration of fly ash. At the time of 270 days, the amount of pores with all diameters (> and <100 nm) surpasses the 180 days. In addition, the amount of pores smaller than 100 nm are nearly the same as compared to that of 90 days, but the amount of pores larger than 100 nm is considerably greater than the 90 days. This phenomenon can be described via a mechanism of progressive leaching of the calcium hydroxide and massive decalcification of CSH hydrates, resulting from water dissolution [37,38]. Consequently, the macroporosity is thought to be increased [39].

**Figure 7:**
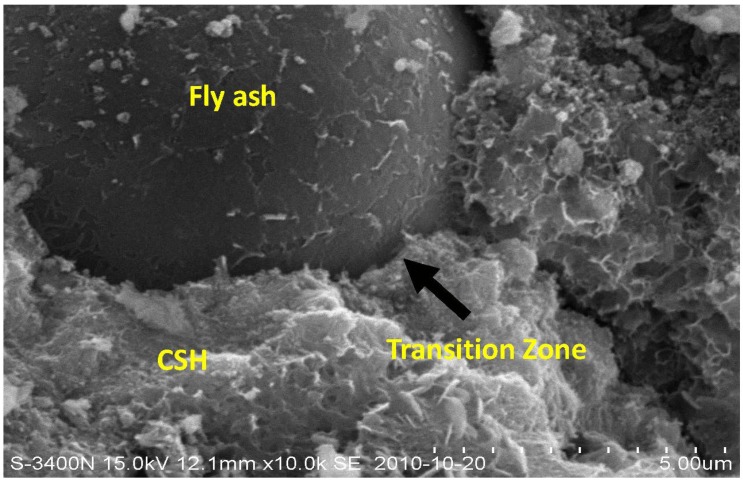
SEM picture of concrete containing 15% fly ash at 90 days of immersion.

Microstructure of concrete CF15 at 90 days of immersion is presented in Figure 7. With the process of hydration, the fly ash particle fills in the space among the calcium silicate hydrate (CSH) gels, which blocks the capillary pores and hence improves the density of concrete. Meanwhile, due to the secondary hydration, the product CSH fills in the micropores of concrete, which results in a denser matrix. It is generally known that permeability and erosion often takes place in the interior pores of concrete. In the process of water transport in concrete, the hydration product gradually dissolves in the water. Given that concrete is rather dense and water-resistant, the rate of permeability is thought to slow down.

## 3. Experimental Section

### 3.1. Materials and Mixture Proportions

#### 3.1.1. Cement

In experimental studies, the P.O 42.5 Portland cement which is produced by Starfish Onoda cement limited company of Shenzhen, China, was used. The chemical properties of this cement are given at Table 5.

**Table 5:** Chemical composition and physical properties of the cement and fly ash.

Item	Cement	Fly Ash (FA)
*Composition (Mass % as Oxide)*		
Calcium oxide (CaO)	64.67	4.74
Silica (SiO_2_)	18.59	62.32
Alumina (Al_2_O_3_)	4.62	23.95
Iron Oxide (Fe_2_O_3_)	4.17	1.33
Magnesium oxide (MgO)	2.35	2.04
Sulfur trioxide (SO_3_)	3.32	1.25
Potassium oxide (K_2_O)	0.92	0.76
Sodium oxide (Na_2_O)	–	–
Loss on ignition (LOI)	1.03	3.12
*Physical Properties*		
Specific surface area (m^2^/Kg)	345	391
80 μm sieving fineness (%)	4.15	8.30

#### 3.1.2. Aggregates

Course aggregate that was obtained from a quarry of AnTuo Mountain, Shenzhen was used in tests. The maximum and the minimum particle sizes of coarse aggregate are 20 mm and 5 mm, respectively. As a result of experiments, the unit weight of the coarse aggregate is 2700 kg/m^3^. River sand was employed as the fine aggregate according to JGJ52-2006 [40] standard, whose fineness modulus is 2.61 and unit weight is 2632 kg/m^3^.

#### 3.1.3. Water

Distilled water was used in concrete production.

#### 3.1.4. Fly Ash

Type I fly ash (FA) was obtained from a power station in Ma Bay of Shenzhen. The chemical composition of fly ash is also provided in Table 1.

#### 3.1.5. NaCl

Sodium chloride that has purity above 99% was used to perform chloride bath for the specimens.

The mixture proportions of concrete are summarized in Table 2. As seen from Table 6, three concrete mixtures were prepared. Concrete without any fly ash was taken as the reference specimen, while the remaining mixtures had two fly ash replacements with the proportions of 15% and 30%, respectively. Water/binder ratio (W/B) was kept constant as 0.47.

**Table 6:** Mix proportions of concrete.

Mix ID	W/B ^a^	Composition (kg/m^3^)				
		Cement	Sand	Crush Stone	Fly Ash	Water
CF0	0.47	409	720	1079	0	192
CF15	0.47	348	697	1054	61	192
CF30	0.47	286	689	1041	123	192

^a^ W/B: water/binder ratio.

### 3.2. Chloride Immersion Test

**Figure 8:**
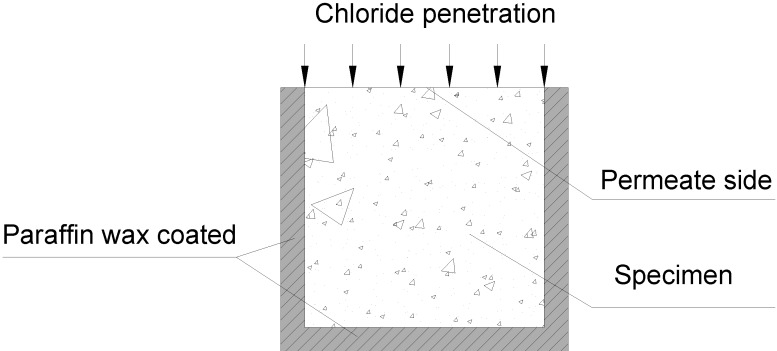
Specimen coated with paraffin wax.

**Figure 9:**
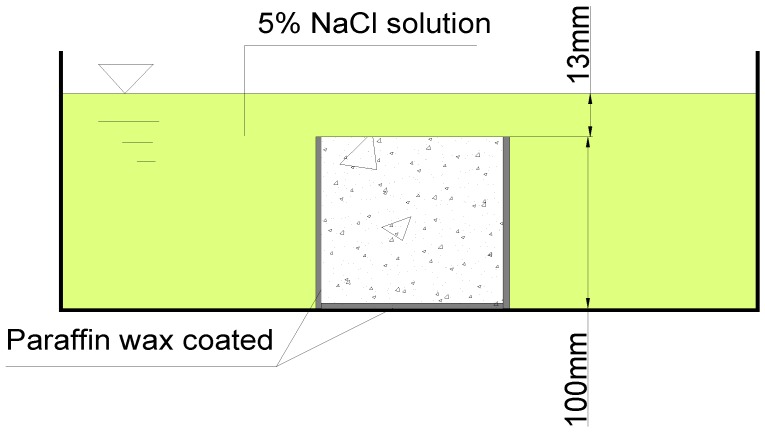
Schematic diagram of 5% NaCl solution immersion of concrete specimen.

The concrete specimens with a size of 100 mm × 100 mm × 100 mm were demoulded after 24 h and cured at the room temperature 20 °C and 95% relative humidity until the test age. In order to have one-directional flow through the concrete, wax coating was applied to the side faces and the bottom face of the specimens before they were completely saturated in the 5% NaCl solution (see Figure 8). The chloride penetration of concrete was tested with the procedures described in AASHTO T259 [41]. The test arrangement for chloride immersion is illustrated in Figure 9. The scheduled period of the immersion was set as 30, 60, 90, 120, and 150 days.

### 3.3. Chloride Ion Concentration Accumulated in the Surface Layer

After the concrete samples were exposed to NaCl solution for a given time, the chloride ion concentration accumulated in the surface layer was determined by methods of Chemical Analysis (CA) that corresponded to JTJ270-98 [42]. The surface layer with a thickness of 3 mm was cut and grinded into powder. The next step was to clear away the coarse particles by 0.63 mm square hole sieve. Acid-soluble extraction was performed afterwards so as to obtain both the free and bound chlorides. Chloride ion concentration in the leachate was measured by automatic potentiometric titrator (Version: Metrohm 809 Titrando, Metrohm, Beijing, China).

### 3.4. Water Permeability of the Surface Layer

Autoclam was used for testing water permeation of the concrete in this experiment. Autoclam tester is able to non-destructively assess the permeability, and the hydroscopicity of the surface area in concrete materials. The tester’s system dates back to the 1980s, and was proposed by Calm. Basheer [43] developed and standardized the whole test system and made it perform automatively. The method exerts an important role in quantifying the durability of concrete served in the aggressive environment. Due to its high accuracy and easy operation as compared to other permeability tests, the Autoclam has been put into service for years. The test system is controlled by a microprocessor and the results data can be analyzed by computer.

**Figure 10:**
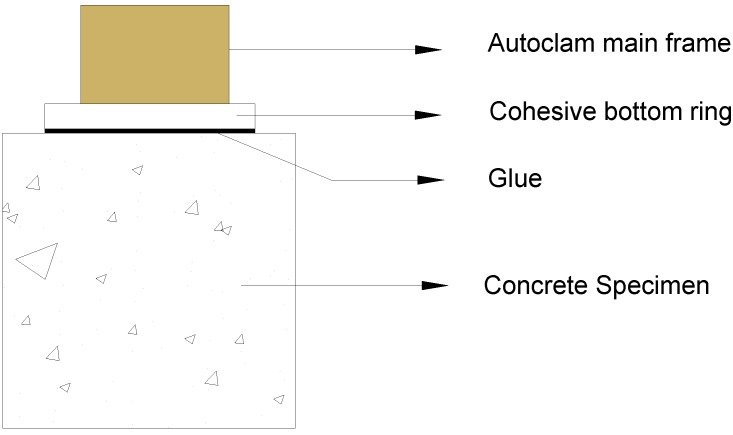
Autoclam permeability test system.

The detailed procedures of this test were in a sequence: The specimens were first immersed in the distilled water until the given period. The specimens were heated in an oven at 80 °C for 12 h prior to the test. After cooling down, the specimens were then performed the water permeability test with tester Autoclam at given intervals of time (1, 2, 3, 4, 5, 6, 7, 8, 9, 10, 11, 12, 13, 14, and 15 min). In this test, each measure was performed on three specimens and the best result was thought to be chosen. When conducting the water permeability test, the surface of the concrete was adhered to cohesive bottom ring (see Figure 10), isolating a test zone with a diameter of 50 mm. The pressure produced by Autoclam main frame is 0.5 bars higher than the air pressure. Based on the results from 5 to 15 min, the volumes of water flow were plotted against square root of time. More details of this method can be found in previous publications [44,45]. The unit of the permeability index is normally 10^−7^m^3^/min^0.5^, and three parallel samples were tested and averaged to get one surface permeability index.

### 3.5. Pore Structure Determination

Mercury intrusion porosimetry (MIP) is widely used to measure the porosity and pore size distribution of cement-based materials for years [46,47]. In this research, the pore size distribution of fly ash concrete was examined by Micromeritics AutoPore IV 9500. In this test, surface layers (with the thickness of around 3 mm from the surface) of concrete specimens were sawed off and crushed (by hammer) into pieces. Then, these pieces were immerged in liquid nitrogen to stop the chemical reaction [48]. Nitrogen adsorption isotherms were measured by a Micromeritics TriSar 3000 Surface Area and Pore Size Analyzer. After elimination of the large aggregates, small pieces (in the range of 300–600 μm) were collected and dried following the solvent (ethanol) replacement drying procedure suggested by Aligizaki in the open literature [49]. The diameter of pores was calculated according to Washburn equation [50], as shown in Equation (1).

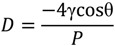
(1)
where *D* is the pore diameter (μm), γ is the surface tension of mercury (mN/m), θ is the contact angle between mercury and the fly ash concrete (°) and *P* is the applied pressure (Mpa). Based on the general suggestions in the literature [51], the surface tension of mercury applied here is 480 mN/m while the contact angle between mercury and fly ash concrete used is 130°. The maximum pressure that could be applied is 30,500 psi (210 MPa), which approximately corresponds to a minimum detectable pore diameter of 6 nm. The Barrett-Joyner-Hanlenda (BJH) method [52], which is still widely used to calculate the mesopore-size distribution, was employed to gain the pore size distribution (PSD) curves.

Scanning Electron Microscopy (SEM) was performed in microscopes. Prior to imaging, the concrete specimens were first crushed into small pieces. These pieces were then placed in a vacuum under 50 °C until constant weight, and coated with a thin gold layer. With the help of SEM results and the MIP data, a more accurate interpretation of pore structure of fly ash concrete can be achieved.

## 4. Conclusions

This study discusses an experimental program carried out to investigate the permeation properties and pore structure of the surface layer of concrete with partial fly ash substitution. The following conclusions can be drawn according to the results of this study:

- In the early immersion period, fly ash has a significant effect on the chloride precipitation in the surface layer. However, the dosage of fly ash has little impact on the chloride precipitation in the surface layer. With the increasing immersion period, amounts of chloride ion in the surface layer of different fly ash contents were nearly the same;
- Fly ash content has a significant influence on the water permeability of the surface layer. With the immersion process, the secondary hydration of fly ash enhances resistance of the concrete to water ingress;
- When a fly ash concrete is under water immersion, both the pozzolanic reaction of fly ash and the water dissolution influence the pore structure development. The pozzolanic reaction is more dominant at the time of 180 days while the water dissolution becomes more evident after 270 days.
